# Factors associated with delaying medical care: cross-sectional study of Nebraska adults

**DOI:** 10.1186/s12913-023-09140-0

**Published:** 2023-02-04

**Authors:** Kendra L. Ratnapradipa, Snehal Jadhav, Josiane Kabayundo, Hongmei Wang, Lisa C. Smith

**Affiliations:** 1 Department of Epidemiology, College of Public Health, University of Nebraska Medical Center, 984395 Nebraska Medical Center, Omaha, NE 68198-4395 USA; 2 Department of Health Services Research & Administration, University of Nebraska Medical Center, Omaha, NE USA; 3 Grace Abbott School of Social Work, University of Nebraska Omaha, Omaha, NE USA

**Keywords:** Health care costs, Transportation, Healthcare utilization, Healthcare delays, Access to care, Rural setting, COVID

## Abstract

**Background:**

Delayed medical care may result in adverse health outcomes and increased cost. Our purpose was to identify factors associated with delayed medical care in a primarily rural state.

**Methods:**

Using a stratified random sample of 5,300 Nebraska households, we conducted a cross-sectional mailed survey with online response option (27 October 2020 to 8 March 2021) in English and Spanish. Multiple logistic regression models calculated adjusted odds ratios (aOR) and 95% confidence intervals.

**Results:**

The overall response rate was 20.8% (*n* = 1,101). Approximately 37.8% of Nebraskans ever delayed healthcare (cost-related 29.7%, transportation-related 3.7%), with 22.7% delaying care in the past year (10.1% cost-related). Cost-related ever delay was associated with younger age [< 45 years aOR 6.17 (3.24–11.76); 45–64 years aOR 2.36 (1.29–4.32)], low- and middle-income [< $50,000 aOR 2.85 (1.32–6.11); $50,000-$74,999 aOR 3.06 (1.50–6.23)], and no health insurance [aOR 3.56 (1.21–10.49)]. Transportation delays were associated with being non-White [aOR 8.07 (1.54–42.20)], no bachelor’s degree [≤ high school aOR 3.06 (1.02–9.18); some college aOR 4.16 (1.32–13.12)], and income < $50,000 [aOR 8.44 (2.18–32.63)]. Those who did not have a primary care provider were 80% less likely to have transportation delays [aOR 0.20 (0.05–0.80)].

**Conclusions:**

Delayed care affects more than one-third of Nebraskans, primarily due to financial concerns, and impacting low- and middle-income families. Transportation-related delays are associated with more indicators of low socio-economic status. Policies targeting minorities and those with low- and middle-income, such as Medicaid expansion, would contribute to addressing disparities resulting from delayed care.

## Background

Limited access to healthcare is a primary public health concern impacting preventative care, health disparities, and overall wellness. Delaying medical care can negatively affect health outcomes, inpatient stays, and the frequency of emergency department visits [1–3]. A paucity of reliable healthcare access increases the likelihood of late-stage diagnoses, higher mortality rates, and poorer survival [4]. Thus, there is a direct correlation between access to healthcare and disease awareness and management [5, 6].

Access to care is in part dependent upon income and distance to healthcare services [4]. The 2010 Patient Protection and Affordable Care Act significantly improved healthcare access, yet cost continues to be a limiting factor [7]. High medical costs and out of pocket expenses have been shown to increase rates of health disparities and preventable deaths among ethnic and minority populations [8–10]. In 2017, 23% of elderly residents in the US reported that cost determined whether health services were sought when sick, prescriptions were filled, or medication skipped. Such choices resulted in avoidable emergency department visits [11]. Like cost of care, transportation is also a leading factor associated with limited healthcare access. Transportation barriers include travel distance, associated time lost in travel, and vehicle access or transit costs [12]. In 2017, 5.8 million individuals in the US delayed medical care due to transportation barriers [11]. Those most affected by transportation issues include minority populations, females, individuals with lower annual incomes, less educated, the elderly, and rural residents [12, 13].

Based on a web survey in June 2020, an estimated 40.9% of US. adults reported delay or avoidance of medical care due to concerns surrounding COVID-19 [14]. This resulted in significant reductions in emergency department visits [15], cancer screenings and treatment [16], and other routine or elective procedures [17]. Although transmission risk was a viable concern, the Centers for Disease Control and Prevention encouraged further research be conducted to explore underlying reasons for delay or avoidance of medical care [14].

Nebraska is a rural, midwestern state with the primary healthcare facilities located in the large urban areas of the eastern part of the state. Indeed, more than 900,000 Nebraskans live in rural, medically underserved areas [18] which can lead to transportation barriers that delay seeking healthcare [19, 20]. In 2020, an estimated 9.3% of Nebraskans age 18 and over required healthcare but could not afford it (i.e., they delayed medical care) [21]. The disparity is even more pronounced when non-Hispanic Black and Hispanic populations are compared to non-Hispanic Whites, 17.1 and 19.9% vs. 8.0%, respectively [21]. Therefore, the purpose of our study was to describe Nebraskans' access to medical care and assess factors associated with ever delaying medical care, ever delaying specifically due to cost or transportation, and delaying medical care within the past 12 months of assessment.

## Methods

Prior to data collection, the study protocol was reviewed by the University of Nebraska Medical Center Institutional Review Board and deemed exempt from oversight. A cover letter sent with the survey provided information for informed consent; informed consent was inferred by completion of the anonymous survey.

### Study design and setting

This anonymous mailed cross-sectional study sampled Nebraska adults (age of majority in Nebraska is 19). Data collection occurred 27 October 2020 through 8 March 2021.

### Sample selection

The only inclusion criteria were a valid mailing address and age 19 or older. To try to ensure racial/ethnic and urban/rural diversity, a stratified sampling design used three levels of county urbanicity (urban large, urban small, rural) with oversamples targeting census tracts that had at least 30% African American (urban large), 30% Native American (rural), or 30% Hispanic (statewide) residents, resulting in six strata used for design weights (Table 1). Oversampling by race/ethnicity was limited to the indicated urbanicity levels due to the unequal geographic distribution of racial groups in Nebraska. The estimated sample size was based on similar studies in Nebraska which assumed a 25 to 28% response rate, or approximately 250 completed responses per strata. The survey was administered through the Bureau of Sociological Research (BOSR) at the University of Nebraska Lincoln. BOSR purchased from Dynata 5,300 Nebraska household addresses selected through the stratified address-based sampling. Addresses were not associated with race/ethnicity of household, so it was not possible to directly identify during sampling whether the oversample was reaching the intended minority populations.

**Table 1:** Sample size and response by strata

Stratum	Surveyed	Responded	Response Rate
Urban large	841	201	23.9%
Urban small	842	207	24.6%
Rural	842	207	24.6%
AA oversample	925	128	13.8%
AI/AN oversample	925	231	25.0%
Hispanic oversample	925	127	13.7%
**Total**	**5300**	**1101**	**20.8%**

### Data collection

The initial mailing included a cover letter (English on one side with Spanish on reverse) requesting the adult with the next birthday after October 1^st^ to complete the survey, a link to an online Qualtrics survey, and a $1 cash incentive. A reminder postcard was sent on 10 November and a follow-up mailing to non-respondents was sent on 1 December containing a cover letter, paper copies (English, Spanish) of the survey, and a postage paid return envelope. A final mailing to non-respondents was sent in early January 2021 consisting of a cover letter, the English version of the survey, and a postage paid return envelope.

Experienced BOSR staff completed data entry and cleaning using double entry of paper returns for quality control. All personal identifying information was removed by BOSR before the final dataset was sent to the researchers for analysis.

#### Instrument

The 8-page questionnaire was developed by the investigators with suggestions from BOSR staff. Content was drawn from the American Community Survey [22], national health [23, 24] and transportation [25, 26] studies and problem lists [27], and peer-reviewed literature [28, 29]. Topic areas included health status, health behaviors, healthcare access and utilization, problem list, telecommunication access, vehicle ownership, transportation, travel distance, and demographics. This analysis was limited to health status, healthcare access and utilization, and demographic variables.

### Measures

#### Health status

A list of yes/no questions of ever diagnosed conditions (heart condition, high blood pressure, diabetes, lung disease, arthritis, stroke, cancer, depression or anxiety disorder) were collapsed into a summary variable of chronic conditions (any versus none).

#### Delayed care measures

Participants were asked a series of yes/no questions related to delayed health care within the past 12 months and other than the past 12 months (due to cost, transportation, unable to get through on the phone, could not get an appointment soon enough, long wait at the office, office closed when arrived, lack of dependent care, could not take time off from work, language barriers). These were combined to create outcome variables of ever delayed, ever delayed due to cost, ever delayed due to transportation, delayed in past 12 months, and delayed in past 12 months due to cost.

#### Demographics

Urban/rural status was based on the 2013 Rural–Urban Continuum Codes for the ZIP code of residence, which was provided directly from BOSR as part of the survey weighting. All other demographic variables were self-reported: year born (calculated age grouped as < 45, 45–64, ≥ 65), gender, Hispanic ethnicity (yes/no), race (mark all that apply: White, Black, American Indian/Alaska Native, Asian/Pacific Islander/Native Hawaiian, prefer not to answer; recoded due to lack of response diversity as White only, Other, and Not reported), marital status (married or partnered, divorced or separated, widowed, single), highest education level completed (< high school graduate, high school diploma or GED, some college, bachelor’s degree or above), health insurance (through employer, purchased on marketplace, Medicare, Medicaid, TRICARE/CHAMPUS, VA, Indian Health Service, Other, None; recoded as any versus none), and annual household income in past year (recoded as < $50,000, $50,000 to $74,999, ≥ $75,000). Another question asked if the participant had one or more individuals that they identified as a personal doctor or health care provider [dichotomized as primary care provider (PCP) yes/no].

### Statistical analysis

BOSR provided a final survey weight, which was used for all analyses. Weighting accounted for address selection probability, nonresponse, within household selection probability, and population age and sex characteristics. Analyses consisted of descriptive statistics, correlation analysis, and logistic regression modeling, with separate models developed for each outcome. Variables were selected using a structured purposeful selection method [30] with initial univariate significance set at *p* < 0.25 to retain a wide range of potential covariates. We also tested for multicollinearity, 2-way interactions, and potential confounding. Due to poor model goodness-of-fit, interaction terms were excluded from final models. We used casewise exclusion for variables with missing data. Significance for 2-tailed testing of final models was set at *p* < 0.05; results are reported as crude odds ratios (OR) and adjusted OR (aOR) with 95% confidence intervals (CI) reported parenthetically. Analysis was conducted using SAS 9.4.

## Results

In total, 1,101 surveys were returned, resulting in an overall response rate of 20.8%. Of the 5,300 selected address, 8.1% (*n* = 430) were deemed ineligible (vacant, no address), and 6.4% (*n* = 341) were undeliverable with unknown eligibility, resulting in an adjusted response rate of 24.3% of delivered surveys. Response rates were lowest for the African American and Hispanic oversamples (Table 1).

Racial and ethnic representativeness are presented in Table 2. Participant characteristics are reported in Table 3. The majority of respondents lived in urban areas (82.7%). Most were female (51.4%), White (89.1%), non-Hispanic (96.4%), married or partnered (69.9%); had a bachelor’s degree or higher (51.7%), annual household income of $75,000 or more (53.9%), some type of health insurance (96.3%); identified a PCP (81.9%); and did not have any chronic health conditions (62.9%). Nearly one-third (31.1%) had current health care bills paid off over time.

**Table 2:** Racial and ethnic representativeness of survey respondents compared to state estimates

		**Unweighted**		**Weighted**	
**Race/Ethnicity**	**Nebraska**^**a**^	**N**	**%**	**N**	**%**
White only	87.7%	893	81.1%	980.5	89.1%
African American only	5.3%	51	4.6%	12.7	1.2%
American Indian/Alaska Native only	1.6%	20	1.8%	5.4	0.5%
Asian/Pacific Islander only	2.9%	8	0.7%	24.9	2.3%
Two or more races	2.4%	17	1.5%	4.1	0.4%
Prefer not to answer or Missing		112	10.2%	73.3	6.7%
**Total**		**1101**		**1101**	
Hispanic (any race)	12.0%	54	4.9%	38	3.5%
Missing		63	5.7%	34	3.00%

^a^Source: https://www.census.gov/quickfacts/NE

**Table 3:** Socio-demographic characteristics of Nebraska adults (Oct 2020-Mar 2021)

Characteristic	Overall unweighted N (%)	Overall weighted N (%)
Rural/ Urban Status		
Rural	462 (42.1)	191 (17.3)
Urban	639 (58.0)	910 (82.7)
Age		
65 years and older	462 (44.9)	193.8 (18.4)
45–64 years	348 (33.8)	375.9 (35.6)
Less than 45 years	219 (21.3)	485.8 (46.0)
Gender		
Male	397 (37.8)	518.2 (48.6)
Female	653 (62.2)	547.5 (51.4)
Race		
White only	893 (81.1)	980.5 (89.1)
Other	96 (8.7)	47.2 (4.3)
African American only	51 (4.6)	12.7 (1.2)
American Indian/Alaska Native only	20 (1.8)	5.4 (0.5)
Asian, Native Hawaiian, Pacific Island only	8 (0.7)	24.9 (2.3)
Two or more races	17 (1.5)	4.1 (0.4)
Not reported	112 (10.2)	73.3 (6.7)
Ethnicity		
Hispanic	54 (5.2)	38 (3.6)
Non-Hispanic	984 (94.8)	1029 (96.4)
Marital Status		
Married or unmarried couple	591 (56.3)	750 (69.9)
Divorced, separated, widowed or never married	459 (43.7)	323 (30.1)
Highest level of education completed		
Bachelor’s or above	366 (34.9)	553 (51.7)
High school or less	342 (32.6)	218 (20.4)
Some college	342 (32.6)	299 (27.9)
Annual Household Income		
< $10,000	51 (5.2)	17 (1.7)
$10,000—$19,999	101 (10.4)	48 (4.8)
$20,000—$29,999	124 (12.8)	63 (6.2)
$30,000—$39,999	102 (10.5)	85 (8.4)
$40,000—$49,999	107 (11.0)	76 (7.5)
$50,000—$74,999	177 (18.2)	177 (17.5)
≥ $75,000	310 (31.9)	545 (53.9)
Insurance		
Any insurance	1040 (95.3)	1056 (96.3)
None	51 (4.7)	40 (3.7)
Access to a primary healthcare provider		
Yes	943 (87.6)	896 (81.9)
No	134 (12.4)	198 (18.1)
Chronic health conditions		
Any condition	266 (24.2)	408 (37.1)
None	832 (75.8)	692 (62.9)
Current health care bills		
No	53 (67.1%)	27.5 (68.9%)
Yes	26 (32.9%)	12.4 (31.1%)

Due to varying degrees of missingness for each variable, not all categories will total to the 1,101 returned surveys. Percentages are reported excluding missing values except when a separate category for non-response is indicated

Over one-third of respondents (37.8%) had ever delayed medical care, 29.7% ever delayed due to cost, and 3.7% ever delayed due to transportation. Additionally, 22.7% delayed care in the past 12 months, 10.1% cost-related.

### Ever delayed care

In univariate analysis, age, race, insurance, and income were significantly positively associated with ever delayed medical care (Table 4). Odds of ever delayed care were 3.21 (1.89–5.45) times greater for those < 45 years and 1.66 (1.02–2.70) times greater for those 45–64 years vs. age ≥ 65. Odds were also higher for those who did not identify only as White (3.49; 1.04–11.74) and those who declined to report race (3.60; 1.46–8.90). Those with no (vs. any) health insurance were 2.77 (1.23–6.26) times more likely to delay; compared to those with annual household income ≥ $75,000 those making $50,000-$74,999 and < $50,000 were 2.22 (1.21–4.10) and 1.96 (1.41–3.36) times more likely to delay care, respectively.

**Table 4:** Crude and adjusted odds ratios (95% confidence intervals) of factors associated with ever delayed medical care, Nebraska, Oct 2020-Mar 2021

Characteristics	Ever Delayed (37.8%)		Due to Cost (29.7%)		Due to Transportation (3.7%)	
	**Univariate**	**Multivariate**	**Univariate**	**Multivariate**	**Univariate**	**Multivariate**
Rural (vs. Urban)	1.09 (0.70, 1.69)	-	1.16 (0.73, 1.86)	-	**2.64 (1.21, 5.77)**	2.17 (0.94, 4.99)
Age group (vs. ≥ 65 years)						
45–64 years	**1.66 (1.02, 2.70)**	**1.78 (1.04, 3.02)**	**2.06 (1.18, 3.60)**	**2.36 (1.29, 4.32)**	0.88 (0.39, 2.02)	-
< 45 years	**3.21 (1.89, 5.45)**	**3.45 (1.90, 6.24)**	**5.18 (2.85, 9.42)**	**6.17 (3.24, 11.76)**	0.42 (0.16, 1.34)	-
Male (vs. Female)	0.70 (0.44, 1.12)	-	0.78 (0.47, 1.30)	-	0.87 (0.40, 1.85)	-
Race (vs. White only)						
Other	**3.49 (1.04, 11.74)**	1.92 (0.67, 5.55)	2.36 (0.81, 6.92)	-	**6.65 (2.09, 21.18)**	**8.07 (1.54, 42.20)**
Not reported	**3.60 (1.46, 8.90)**	1.59 (0.62, 4.09)	1.56 (0.75, 3.24)	-	**3.62 (1.29, 10.15)**	2.60 (0.73, 9.29)
Hispanic (vs. Non-Hispanic)	1.28 (0.50, 3.27)	-	1.57 (0.60, 4.15)	-	2.15 (0.79, 6.02)	-
Marital status (Divorced, separated, widowed, or never married vs. married or unmarried couple)	1.40 (0.86, 2.29)	-	**1.79 (1.07, 3.02)**	0.86 (0.43, 1.79)	2.01 (0.97, 4.17)	-
Education (vs. Bachelor’s degree or above)						
High school or less	1.19 (0.63, 1.97)	-	1.19 (0.63, 2.23)	-	**8.47 (2.06, 34.77)**	**3.06 (1.02, 9.18)**
Some college	1.22 (0.72, 2.08)	-	1.33 (0.76, 2.34)	-	**10.82 (2.65, 44.25)**	**4.16 (1.32, 13.12)**
Health insurance (None vs Any)	**2.77 (1.23, 6.26)**	2.34 (0.84, 6.51)	**3.99 (1.76, 9.06)**	**3.56 (1.21, 10.49)**	1.27 (0.43, 3.75)	-
Income (vs. ≥ $75,000)						
$50,000 to $74,999	**2.22 (1.21, 4.10)**	**2.35 (1.24, 4.43)**	**2.65 (1.37, 5.10)**	**3.06 (1.50, 6.23)**	3.49 (0.51, 23.88)	1.89 (0.35, 10.62)
< $50,000	**1.96 (1.41, 3.36)**	**2.17 (1.16, 4.07)**	**2.03 (1.12, 3.69)**	**2.85 (1.32, 6.11)**	**19.86 (3.78, 104.19)**	**8.44 (2.18, 32.63)**
Access to a primary care provider (No vs. Yes)	1.19 (0.60, 2.39)	-	1.64 (0.81, 3.34)	-	**0.29 (0.10, 0.81)**	**0.20 (0.05, 0.80)**
Chronic health condition (Any vs. None)	1.03 (0.62, 1.70)	-	0.97 (0.56, 1.67)	-	**4.94 (1.47, 16.64)**	3.27 (0.83, 12.86)

Bold indicates *p* < .05

Only age group and income remained statistically significant in the adjusted model. Compared to older adults, those < 45 (aOR 3.45; 1.90–6.24) and 45–64 years (aOR 1.78; 1.04–3.02) were more likely to report ever delaying care. Compared to those who made ≥ $75,000, individuals with incomes $50,000-$74,999 and < $50,000 were 2.35 (1.24–4.43) and 2.17 (1.16–4.07) times more likely to ever delay care.

### Ever delayed care due to cost

Age, marital status, health insurance, and income were significantly associated with ever delaying care due to cost in univariate analysis. Compared to older adults, those < 45 and aged 45–64 were more likely to ever delay due to cost (OR 5.18; 2.85–9.42 and OR 2.06; 1.18–3.60, respectively). Odds were also higher for those who were not married or living as a couple (OR 1.79; 1.07–3.02), those without health insurance (OR 3.99; 1.76–9.06), those who made < $50,000 (OR 2.03; 1.12–3.69) and $50,000-$74,999 (OR 2.65; 1.37–5.10).

After adjustment, age, insurance status, and income remained significant. Compared to older adults, those < 45 and those 45–64 had higher odds of delaying due to cost (aOR 6.17; 3.24–11.76 and aOR 2.36; 1.29–4.32). Those with no health insurance were at higher likelihood of delay due to cost (aOR 3.56; 1.21–10.49), as were those with incomes < $50,000 (aOR 2.85; 1.32–6.11) and $50,000-$74,999 (aOR 3.06; 1.50–6.23).

### Ever delayed care due to transportation

In univariate analysis, rural (vs. urban; OR 2.64; 1.21–5.77), Other race (vs. White only; OR 6.65; 2.09–21.18) and race not reported (OR 3.62; 1.29–10.15), education of high school or less (OR 8.47; 2.06–34.77) and some college (OR 10.82; 2.65–44.25) compared to bachelor’s degree or above, income < $50,000 (vs. ≥ $75,000; OR 19.86; 3.78–104.19), and having any chronic health conditions (vs. none; OR 4.94; 1.47–16.64) were associated with higher odds of ever delaying care due to transportation, while those who did not identify a PCP were 71% less likely to have ever delayed due to transportation (OR 0.29; 0.10–0.81).

In the final model, race other than White only (aOR 8.07; 1.54–42.20), education of high school or less (vs. bachelor or above; aOR 3.06; 1.02–9.18) and some college (aOR 4.16; 1.32–13.12), income < $50,000 (vs. $ > 75,000; aOR 8.44; 2.18–32.63), and not identifying a PCP (aOR 0.20; 0.05–0.80) remained significant.

### Delayed care in the past 12 months

The univariate analysis showed that age, marital status, income, and current healthcare bills paid off over time were significantly associated with delayed care in the past 12 months (Table 5). Compared to older adults, the odds of delayed care in the past year were 2.53 (1.39–4.61) for age < 45 and 1.77 (1.00–3.15) for ages 45–64. Individuals who were divorced, separated, widowed, or never married were 2.29 (1.34–3.91) times more likely to report delayed care in the past year than married or unmarried couples. Compared to those earning ≥ $75,000, those making $50,000-$74,999 and < $50,000 were 2.75 (1.35–5.62) and 3.63 (1.94–6.78) times more likely to delay care in the past year, respectively. Those with current healthcare bills paid off over time were 3.15 (1.17–5.60) times more likely to delay care in the past year than those who did not.

**Table 5:** Crude and adjusted odds ratios (95% confidence intervals) of factors associated with delayed medical care in past year, Nebraska, Oct 2020-Mar 2021

Characteristics	Delayed Care (22.7%)		Due to Cost (10.1%)	
	**Univariate**	**Multivariate**	**Univariate**	**Multivariate**
Rural (vs. Urban)	0.99 (0.6, 1.64)	-	1.38 (0.69, 2.78)	-
Age group (vs. ≥ 65 years)				
45–64 years	**1.77 (1.00, 3.15)**	1.76 (0.87, 3.55)	**4.8 (2.04, 11.29)**	**4.39 (1.66, 11.58)**
< 45 years	**2.53 (1.39, 4.61)**	**2.86 (1.39, 5.86)**	**9.85 (4.14, 23.48)**	**14.24 (4.79, 42.35)**
Male (vs. Female)	0.74 (0.44, 1.25)	-	0.93 (0.44, 1.96)	-
Race (vs. White only)				
Other	2.05 (0.64, 6.52)	-	3.85 (0.95, 15.63)	-
Not reported	1.43 (0.69, 2.97)	-	1.22 (0.55, 2.72)	-
Hispanic (vs. Non-Hispanic)	2.03 (0.75, 5.46)	-	**3.97 (1.26, 12.48)**	1.68 (0.59, 4.82)
Marital status (Divorced, separated, widowed, or never married vs. married or unmarried couple)	**2.29 (1.34, 3.91)**	1.34 (0.67, 2.66)	**4.22 (2.04, 8.75)**	1.63 (0.67, 3.96)
Education (vs. Bachelor’s degree or above)				
High school or less	1.33 (0.69, 2.56)	-	**3.65 (1.3, 10.21)**	1.74 (0.52, 5.78)
Some college	1.75 (0.97, 3.15)	-	**4.34 (1.75, 10.74)**	1.68 (0.61, 4.61)
Health insurance (None vs Any)	1.66 (0.73, 3.76)	-	**4.66 (1.93, 11.27)**	1.40 (0.52, 3.80)
Income (vs. $75,000 +)				
$50,000 to $74,999	**2.75 (1.35, 5.62)**	2.20 (0.98, 5.00)	**6.83 (1.97, 23.7)**	3.38 (0.83, 13.84)
< $50,000	**3.63 (1.94, 6.78)**	**3.41 (1.52, 7.64)**	**13.58 (4.4, 41.92)**	**9.60 (2.49, 37.05)**
Access to a primary care provider (No vs. Yes)	1.45 (0.70, 2.97)	-	2.09 (0.87, 5.05)	-
Chronic health condition (Any vs. None)	1.47 (0.82, 2.64)	-	2.03 (0.71, 5.78)	-
Current health care bills (Yes vs. No)	**3.15 (1.77, 5.60)**	**2.54 (1.37, 4.73)**	**6.01 (2.8, 12.9)**	**5.26 (2.35, 11.78)**

Bold indicates *p* < .05

In the adjusted model, only age, income, and current healthcare bills remained statistically significant. Compared to older adults, those < 45 (aOR 2.86; 1.39–5.86) were more likely to report delayed care in the past year. Overall, income was significant only when comparing ≥ $75,000 to those making < $50,000 (aOR 3.41; 1.52–7.64). Lastly, outstanding healthcare bills was significant (vs. none; aOR 2.54; 1.37–4.73).

### Delayed care in the past 12 months due to cost

The univariate analysis found age, ethnicity, marital status, education level, insurance status, income, and current pending healthcare bills to be statistically significant. The results show that the adults ≤ 45 (OR 9.85; 4.14–23.48) and 45–64 (OR 4.8; 2.04–11.29), Hispanics (vs. non-Hispanics; OR 3.97; 1.26–12.48), never married (OR 4.22; 2.04–8.75), education of high school or less (OR 3.65; 1.3–10.21) and some college (OR 4.34; 1.75–10.74), no insurance (vs. any; OR 4.66; 1.93–11.27), income < $50,000 (vs. ≥ $75,000; OR 13.58; 4.4–41.92), and current health care bills (vs. none; OR 6.01; 2.8–12.9) were associated with higher likelihood of delayed care due to cost in the past year.

After adjustment, adults ≤ 45 (aOR 14.24; 4.79–42.35) and 45–64 (aOR 4.39; 1.66–11.58), income < $50,000 (aOR 9.6; 2.49–37.05), and current healthcare bill (aOR 5.26; 2.35–11.78) were statistically significant for delayed care due to cost in the past year.

## Discussion

The objectives of this study were to describe Nebraskans' access to health care and examine factors associated with delayed care (ever and past 12 months). Delayed medical care is a public health concern due to its association with negative health outcomes and increased costs [1]. Our findings show that 37.8% of respondents had ever delayed care, and 22.7% delayed in the past 12 months. Cost was the most common reason for ever delaying care, accounting for 78.6% of delayed care. Transportation-related delay (3.7%) was higher than the 1.8% national average reported by Wolfe and colleagues [11]. In addition to cost and transportation, age and household income were significantly associated with delayed care. Our access measures such as any insurance differ slightly from state estimates based on the 2020 Behavioral Risk Factor Surveillance System (96.3% vs. 87.9%, respectively) [31].

Our results show that age is a significant predictor of ever delayed medical care, which is a measure of lifetime prevalence. Since more opportunities are available for delayed care in the elderly, we expected that older age would have higher lifetime prevalence, as opposed to recent (past 12 month) prevalence. Such opportunities are due to life longevity and the putative need for more medical care in older age. Instead, the odds of both ever delayed care and delayed in past 12 months were much higher for the youngest age group. These results are consistent with previous research on delays in the past 12 months, which found younger individuals are more likely to delay seeking medical care than the elderly [32, 33]. The rates of healthcare utilization among young adults may be a response to a paucity of health insurance and increased healthcare costs [34, 35], although our sample had low prevalence of no insurance. Conversely, it may also reflect better overall health among younger individuals, and therefore unprioritized routine or preventive care. Alternatively, our results may partially reflect question wording rather than true lifetime prevalence. We combined responses for questions asking about delays in the past 12 months and other than the past 12 months for a variety of causes. Delaying prior to the past year would be subject to increased recall bias and was not asked in as much detail as delays in the past 12 months.

Our study supports prior research conducted globally and in the United States that cost is a primary barrier to medical care [36, 37]. In the US, an estimated 6.3% of adults delayed medical care due to costs in 2019–2020 [38] and 9.3% of Nebraskans delayed due to cost in 2020 [21], which is lower than the 10.1% in our study conducted in late 2020. We also estimated that 28.4% of Nebraska adults delayed care due to cost other than the past year, for an overall lifetime prevalence of 29.7%. Prior research identified that people with lower income, uninsured, underinsured, and who have other healthcare bills have higher rates of delaying care because of cost [1, 39]. For example, a study conducted nearly 30 years ago found that the odds of delaying care for participants who were uninsured and with lower incomes was 12 times greater than the odds of other patients [1]. In our study, factors associated with cost-related delays included younger age, lower income, and lack of insurance. Our results are at a lower magnitude than those reported by Weissman, and we adjusted for a number of socioeconomic factors. Further, these results likely reflect, at least in part, the devastating impacts of the COVID-19 pandemic in terms of healthcare and economics. Routine and nonemergency medical care was avoided in response to lost employment, wages and insurance. As such, this environmental context may exacerbate the disparities identified in the study and provide insight for future healthcare management.

Medical billing is complex, and health insurance (public or private) typically does not cover all healthcare-related costs. Households earning up to 138% of the federal poverty level qualify for Medicaid in Nebraska ($30,305 for a family of 3 in 2021) [40]. In our study, those who made $50,000 to $74,999 actually had higher odds of delay due to cost than those who made less than $50,000. This may reflect economic difficulties of those earning above the thresholds to qualify for assistance. In line with prior research, uninsured participants in our study were more likely to report delayed medical care due to cost compared to insured participants. We should note that our definition of insurance included government programs including Medicaid and Medicare, so relatively few respondents lacked any type of insurance. Considering that an estimated 9.7% of US adults do not have any health coverage, and that Hispanics are more likely to be uninsured compared to other groups [1], cost-related delays should continue to be a focus of disparities research.

We also examined delays specifically due to transportation. Less than 8% of respondents, weighted to represent 3.7% of the Nebraska adult population, had ever delayed due to transportation issues, which resulted in wide confidence intervals in our analysis. Transportation barriers varied by education level, income, and race in adjusted analysis. Access to reliable transportation can be expensive (e.g., vehicle purchase price, maintenance, gas, licensing and registration, and insurance for private vehicles), so it is not surprising that those in the lowest income category were more likely to experience transportation-related healthcare delays compared to those in the highest income category. Interestingly, although odds ratios comparing education to those with a bachelor’s degree or above were significant for both comparison groups, those with some college were 416% more likely to have transportation-related delays, while those with high school or less were 306% more likely. Race other than White had more than 8 times higher odds of transportation-related delays, although the lack of precision in our racial categories makes interpretation difficult. Previous studies identified transportation barriers including lack of vehicle access, long distances to the healthcare providers, and transportation cost [12]. The shortest distance between the clinic and the patient has been associated with a higher likelihood of maintaining the healthcare appointment [41], indicating distance is an important aspect of transportation. Transportation barriers are more likely to be reported by those with less education, lower income, racial and ethnic minority groups [12, 13, 20, 41], socially disadvantaged individuals, those lacking a family member or friend to support them, and individuals who live in rural medically underserved areas [13, 19, 42].

Strengths of this study include the statewide representative sample of the adult population in Nebraska and detailed questions about delayed access at different time points and for differing reasons. However, we were unable to stratify our analysis by race as originally planned due to the lack of diversity in our sample. Although we oversampled in areas with higher percentages of minorities, Nebraska lacks racial diversity in general and we were unable to weight by racial category. Additionally, one of our outcomes (delay due to transportation) had low prevalence resulting in wide confidence intervals. We classified delayed care in the past year as well as other than the past year to create an ever-delayed response, but this may have been subject to recall bias and potential misclassification. Additionally, our study was cross-sectional and we cannot make causal assumptions. Many of the sociodemographic variables in our models are time-dependent and may differentially impact healthcare access over time.

Access to healthcare is a social determinant of health. We examined one aspect of access – delayed care – and found that income level was the only factor we studied that was associated with delay for each reason (unspecified, cost, transportation). Delayed medical care is complex in its underlying origins; more study of sociodemographic interacting factors is needed to identify viable interventions tailored to different group needs. For many health conditions, early identification and treatment may have prolonged and profound health consequences by limiting progression and damage. Public health interventions and policies that aim to support those with transportation barriers, uninsured, underinsured, and among racial and ethnic disadvantaged groups would contribute to addressing disparities resulting from delayed care. Future study examining how changes in Medicaid expansion coverage (implemented in Nebraska at the time this study was conducted) impact self-reported delayed care is warranted.

## Conclusions

Healthcare access and utilization is a known social determinant of health that can have profound consequences across primary, secondary, and tertiary prevention efforts. Although the Affordable Care Act and expansion of Medicaid coverage has attempted to address some of the cost-related issues, significant gaps remain. Our study, conducted toward the end of the first year of the COVID pandemic, highlights continued struggles with delayed care in Nebraska. Delayed care affected more than one-third of Nebraskans, primarily due to financial concerns, and impacting low- and middle-income families. Transportation-related delays are associated with more indicators of low socio-economic status. Policies targeting minorities and those with low- and middle-income, such as Medicaid expansion, would contribute to addressing disparities resulting from delayed care.

## Data Availability

The dataset used and analyzed during the current study are available from the corresponding author on reasonable request.
